# Variable-Structure Proportional–Integral–Derivative Laser Solder Joint Temperature Intelligent Control Method with Adjustable Power Upper Limit

**DOI:** 10.3390/mi14081618

**Published:** 2023-08-17

**Authors:** Mingchao Li, Pengbin Cao, Cong Zhang, Kuan Yan, Yuquan Zhang

**Affiliations:** 1 School of Mechanical and Electrical Engineering, Wuhan Institute of Technology, Wuhan 430205, China; 2 Wuhan Fiberhome Technical Services Co., Ltd., Wuhan 430205, China

**Keywords:** laser soldering, electronic assembly, solder joint temperature, adjustable power upper limit, variable-structure PID, parameter optimization

## Abstract

Laser soldering is a crucial soldering technique in the realm of electronic assembly. The temperature of the solder joint is intimately connected with the quality of the solder. This paper introduces an adjustable power upper limit variable-structure Proportional–Integral–Derivative (PID) intelligent control method for regulating the temperature of the solder joint during laser soldering. Distinct laser power limits are employed for workpieces with varying heat capacities. The solder joint temperature is monitored through an infrared thermometer, which enables closed-loop temperature control via a variable-structure PID algorithm. Residual neural network (ResNet) models are utilized to predict key soldering process parameters. This method has been executed and validated on a practical testing platform. Compared to other laser soldering control techniques, the proposed method demonstrates a low overshoot, rapid dynamic response, and swift adjustment capabilities, effectively enhancing the soldering quality and production efficiency.

## 1. Introduction

Laser soldering is extensively utilized in electronic module assembly. It has minimal thermal effect areas, with the minor focal-spot diameter reaching up to a micron, thus preventing issues such as solder bridging during soldering [1,2]. During the soldering process, the temperature of the solder joints is a critical factor that influences the reliability of the solder [3] and is closely associated with quality indicators like the wettability of the solder joints [4]. Nevertheless, controlling the process can be challenging due to the high energy density of the laser. If not properly controlled, it can result in damage to electronic components and circuit boards or even lead to poor soldering. Different combinations of parameters also impact the quality [5], making it essential to select appropriate parameters. It is plausible that the optimal parameters may vary among different materials being soldered. The primary reason for this is the differing properties of the materials, as well as their distinct thermal diffusion and thermal capacity. Consequently, establishing an intelligent control method for the solder temperature is crucial to enhancing the performance of laser soldering.

The temperature control of solder joints is primarily achieved by adjusting the laser power to align the solder joint temperature with the target temperature. However, due to the rapid heating speed of laser heating, the performance requirements for the controller, actuator, and temperature feedback unit within the system are elevated. Low-performance hardware facilities cannot fully meet the demand, while high-performance hardware facilities increase the costs. To address this issue, scholars have established models to predict heating temperatures [6,7,8]. Among them, Zhihua Chen et al. [9] proposed a thermodynamic model through which the parameters involved in the controller can be calculated to anticipate temperature changes during laser soldering. Compared with traditional control methods, this scheme has obvious advantages. Unfortunately, this method combines open-loop control, and the parameters need to be obtained through modeling, resulting in certain limitations when soldering different types of workpieces.

Compared with the open-loop strategy, the PID algorithm, as one of the commonly used closed-loop control algorithms, has been widely used in many control systems [10,11,12]. Numerous scholars have improved the traditional PID algorithm based on the specific characteristics of the actual controlled objects. Fathi et al. [13] designed a feedforward PID controller to adjust the scanning speed in laser additive manufacturing. A new knowledge-based Hammerstein model was proposed, which includes linear dynamic and nonlinear memory-less blocks, and its parameters were identified offline using experimental data. The architecture of the control system consists of a PID controller and a feedforward module. The results showed that the controller has a lower overshoot and faster response time. The PID algorithm can also be used in motor control by employing the Symbiotic Organisms Search (SOS) algorithm and Stochastic Fractal Search (SFS) for PID parameter optimization to enhance system performance. These bio-inspired algorithms are lauded for their efficiency and lack of tuning requirements [14,15]. In the dual-beam laser welding platform constructed by Yu Huang et al. [16], an independent error compensation control method based on an adaptive fuzzy PID controller is utilized to compensate for welding deviations in real time, enabling the tracking of both sides of the weld seam. The parameters are adjusted online according to the observed error and its changing rate. This makes the controller more robust so that it can deal with different welding conditions.

Prabhakar G. et al. [17] designed a fuzzy proportional–derivative (PD + I) controller for automobile control systems, which provides adaptive capabilities in setpoint tracking performance. Based on performance measures such as the integral absolute error (IAE) and integral squared error (ISE), the proposed fuzzy (PD + I) structure shows superior performance in servo and regulatory problems. This controller avoids the integral windup problem and suppresses the derivative kick. Choon Lih Hoo et al. [18] discussed the effect of integrating derivative control on the Steady-State Integral–Proportional–Integral Controller (SIPIC) in motor-speed control. The experimental results showed that the SIPIC + D controller has better speed control on a direct current motor compared with other existing anti-windup methods with added derivative control.

In addition, process parameters such as the heating time and temperature are closely related to quality. And, due to the strong coupling of parameters and the multi-mode influence of these parameters on processing indicators, it is a challenge for engineers to determine the parameters that meet the requirements. Some studies [19,20,21] used a convolutional neural network (CNN) to process welding images to automatically determine the appropriate welding process parameters. For instance, in laser welding, Alex Božič et al. [22] established a remote laser welding system based on a CNN, which primarily recognizes and tracks the welding effect through a camera and controls the laser output power using a PID controller. Even so, this laser processing method optimized with a CNN is mainly suitable for laser brazing, which does not require temperature feedback and is not appropriate for selective soldering of precision devices. However, the use of a CNN instead of traditional process-parameter tuning methods holds a certain reference value. Zhong Yuguang et al. [23] proposed integrating the gray correlation model (GCM), genetic algorithm simulated annealing (GASA), and artificial neural network (ANN) algorithms to solve the prediction problem of process parameters. By using the weights and thresholds of the GASA neural network, global optimization can be achieved within a relatively short training time. The original network is simplified by utilizing the GCM as a preprocessing tool. As the number of network layers increases, the problem of gradient vanishing or diminishing arises. In order to address the issues of information loss and degradation caused by model degradation, He et al. [24] proposed using a ResNet. The ResNet utilizes residual learning units and introduces a shortcut mechanism, enabling the deep application of a CNN [25]. Chunyang Xia et al. [26] investigated and compared the classification performance of several notable CNN architectures, such as ResNet, EfficientNet, VGG-16, and GoogLeNet, within experiments. The findings revealed that the model group employing the ResNet architecture achieved the highest classification accuracy. Junmyoung Jang et al. [27] utilized a ResNet to analyze the microstructures of weldment produced using carbon steel to measure the fraction of acicular ferrite. The model is trained to distinguish acicular ferrite from the microstructures of dataset images and then estimate its accuracy.

In order to enhance the effectiveness of laser soldering and simplify the process of setting process parameters, this paper establishes a laser soldering system primarily composed of laser, solder joint temperature detection, and solder joint quality detection. The proposed laser soldering system significantly differs from traditional laser soldering systems. First, a laser power control method with an adjustable upper power limit is proposed to improve workpieces’ adaptability with varying heat capacities. It can also weaken the derivative kick phenomenon. Second, a high-speed infrared thermometer is employed to provide feedback on the solder joint temperature, and a variable-structure PID algorithm is introduced to control this temperature. By setting the appropriate error value and limiting the integral value, the integral windup phenomenon of the controller can be suppressed. Third, in order to streamline the process of setting parameters and further enhance the soldering quality, an optimization model of the parameters based on a ResNet is designed. This approach can circumvent numerous experiments, reduce the soldering research cycle, and hold great significance for improving the application value of laser soldering and simplifying the soldering process.

## 2. Experimental Setup

### 2.1. Task Analysis

In laser soldering technology, the temperature of the solder joint is a pivotal factor. It is essential for ensuring an appropriate wetting angle, good adhesion, and the formation of a robust solder joint. Various parameters influence the solder joint temperature, including the laser power, heating time, and solder material.

If the temperature of the solder joint fails to reach the target temperature, the solder may not fully melt, leading to poor wetting and incomplete formation of the solder joint. Due to thermal cycling or mechanical stress, the solder joint may weaken or fail over time. On the other hand, if the temperature of the solder joint exceeds the target temperature, the electronic components being soldered may overheat, leading to oxidation, deformation, or even melting, which could also result in a decline in the quality of the solder joint [28]. Therefore, controlling the temperature of the solder joint is critically important in the laser soldering process.

When the laser acts on the workpiece, the heat source generated by the laser can be treated as the surface heat source, which is set as the heat flow on the surface of the workpiece and the solder. The heat distribution formula is

(1) $$q(x,y)=\frac{2AP}{\pi w^{2}}\exp\left(-2r^{2}/w^{2}\right)$$

Table 1 presents an explanation of the symbols used in the formula. According to Equation (1), the surface temperature of the workpiece principally relies on the laser power at an equivalent distance from the laser center. In the solder joint temperature control process, the primary objective is to sustain the surface temperature of the workpiece at the target temperature by regulating the laser power.

### 2.2. Control System of Laser Soldering

**Figure 1:**
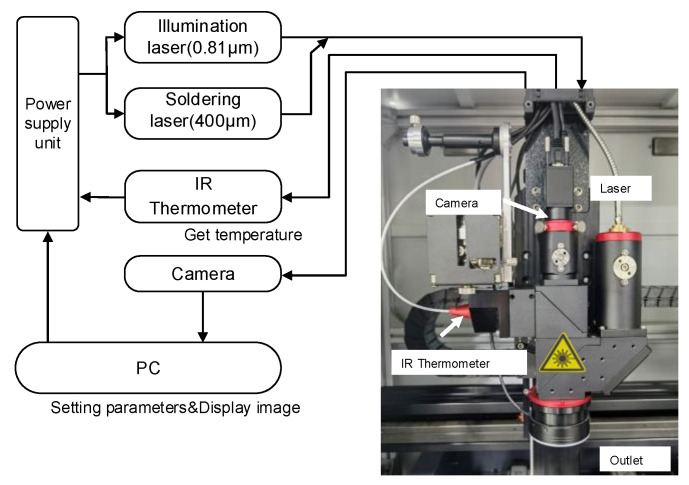
Laser soldering system.

**Figure 2:**
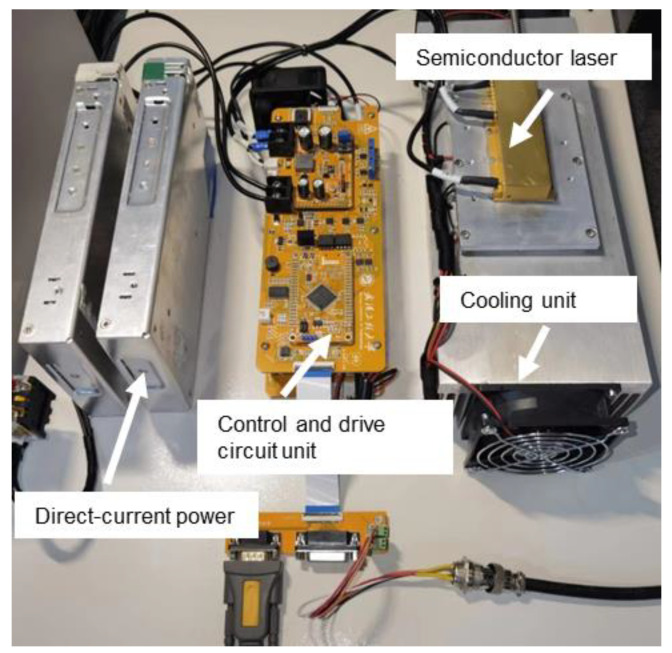
Power supply unit with integrated controller and driver for the semiconductor laser.

The structure of the laser soldering system is shown in Figure 1. It mainly consists of a power supply unit with an integrated controller and a driver for the semiconductor laser (as shown in Figure 2, which mainly includes a drive and control unit, a high-power fiber-coupled semiconductor laser, and its cooling unit), a high-speed infrared thermometer, a five-light coaxial optical lens (including a camera), a three-axis motion platform, and a host computer. The power supply unit with the integrated controller and driver for the semiconductor laser is a control system based on ARM, which is mainly used to collect the solder joint temperature signals of the high-speed infrared thermometer, receive the parameter commands set by the host computer, and run the temperature control algorithm. The host computer is mainly used to set the process parameters, run the neural network algorithm, and control the three-axis motion platform.

During the soldering process, a high-speed infrared thermometer is primarily used to feedback the surface temperature of the solder joint, and a constant current circuit with a rapid current response capability is used to drive the semiconductor laser. Due to the rapid heating of the solder by the laser, if a traditional PID structure is adopted, it places high demands on the command cycle of the controller, temperature sampling speed, and current response time of the semiconductor laser driver circuit. At the same time, it cannot effectively resist random disturbances during system operation. Considering the phase change of the solder and the non-linear and time-varying surface temperature of the solder joint during the soldering process, there should be good adaptability for workpieces with different heat capacities. This places higher requirements on the robustness and adaptability of the controller. Therefore, designing a suitable method for controlling the temperature of the solder joint is crucial to improving the performance of laser soldering.

The ResNet is employed to identify and analyze pre-soldering joints, thereby determining the optimal upper limit of the laser power and heating time. Deep learning has emerged as a prevalent method in machine vision and pattern recognition owing to its strengths in feature learning and image classification. Within learning, the CNN stands out as one of the most popular algorithms, having been successfully applied to object detection, action recognition, and image classification [29,30]. The gradient-weighted class activation mapping (Grad-CAM) technique [31] and the confusion matrix [32] can be employed to ascertain the cause of errors and evaluate the effectiveness of the classification during the classification process.

Considering the characteristics of laser soldering heating, this study proposes selecting an appropriate upper limit value of the laser power for varying workpieces. Subsequently, combining the variable-structure PID controller and ResNet can enhance the solder joint temperature control accuracy and streamline the process parameter configuration. The experimental results demonstrate that the proposed system exhibits satisfactory temperature tracking performance for different workpieces, achieving a minimum solder joint temperature error of ±1.85 °C and an intelligent process parameter matching accuracy rate of 90.41%. In electronic assembly, this approach can significantly improve the soldering quality of precision devices, yield rates, and processing efficiency.

## 3. Strategies for Controlling Solder Joint Temperature

### 3.1. Variable-Structure PID with Adjustable Upper Limit of Power

Due to the complex structure of the control object in laser soldering, this study proposes utilizing different upper limits of the laser power based on the specific workpieces. Simultaneously, a variable-structure PID controller is introduced to address the issues of low-temperature control accuracy and poor adaptability in existing solder joints during laser soldering. The subsequent sections present a theoretical derivation of the method.

#### 3.1.1. Improved PID Algorithm

The traditional PID equation is represented by Equation (2). However, neither the PID parameter self-tuning algorithm nor the techniques of integral separation, integral limiting, variable speed integration, and incomplete differentiation altered the structure of the traditional PID control algorithm formula. Theoretical analyses and extensive practical applications indicate that such controllers struggle to simultaneously fulfill the requirements of tracking reference input and suppressing disturbances, often resulting in trade-offs between responsiveness and overshoot, as well as static and dynamic performance.

(2) $$u(n)=K_{p}e(n)+K_{i}\int e(n)dn+K_{d}\frac{de(n)}{dn}$$

Since the amplitude limit within the system can cause the output of the controller to saturate, the integral term may play a counteracting role at this point. This can result in a significant overshoot and an extended system adjustment time, potentially leading to system instability. Concurrently, when the solder transitions to a liquid state, various properties, such as thermal conductivity, specific heat capacity, and melting thermophysical characteristics, will undergo alterations. These changes may influence the solder joint temperature distribution and heat conduction attributes, thus impacting the effectiveness of temperature control. Consequently, it is essential to improve the control strategy.

Given the shortcomings of the traditional control method, the variable-structure PID is introduced to control the temperature of the solder joint of laser soldering in a closed loop, which is formulated as follows (Table 2 provides the symbol explanation for the control algorithm formula):

(3) $$u(n)=\left\{\begin{array}{ll} K_{p}e(n) & |e(n)|⩾E_{A}\\ K_{p}e(n)+K_{d}\frac{de(n)}{dn} & E_{B}⩽|e(n)|<E_{A}\\ K_{p}e(n)+K_{i}G_{i}(e)\int e(n)dn+K_{d}G_{d}(e)\frac{de(n)}{dn} & |e(n)|<E_{B} \end{array}\right.$$

**Figure 3:**
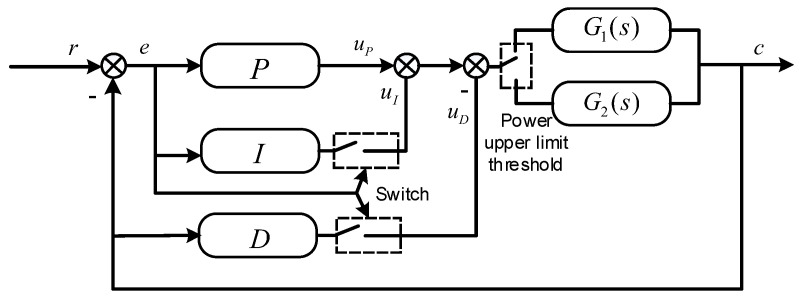
Variable-structure PID with adjustable upper power limit.

As depicted in Figure 3, a variable-structure PID diagram with an adjustable power upper limit is presented. Building upon the traditional PID model, proportional, integral switching and an upper power limit selection unit have been incorporated. The control strategy varies based on the magnitude of the error:

- For the most significant errors, P control accelerates the response speed.
- For significant errors, PD mode mitigates overshoot.
- For minor errors, PID mode eliminates steady-state deviation.

When the computational result of the controller exceeds the set upper power limit, the final output laser power will be maintained at the set power upper limit. Conversely, the original value will be maintained if the computational result exceeds the upper power limit. By setting an upper power limit, the heating rate can be controlled. If the heating rate is too fast, it can cause the flux inside the solder to boil, leading to the spattering of the solder (tin bead).

When utilizing a variable-structure PID, P control is employed in Stage $e(n)\geq E_{A}$ (where errors are large) to facilitate a rapid response and circumvent the influence of integral saturation on the system. During the intermediate process in Stage $E_{B}\leq |e(n)|<E_{A}$, the PD control mode is applied, using the differential component to diminish system overshoot while maintaining a swift response. In Stage $|e(n)|<E_{B}$, which features small deviations, the integral action progressively increases as error e(n) decreases, and the differential action gradually decreases, effectively eliminating steady-state bias. By monitoring the temperature differential and the output value of the controller and taking into account the saturation state of the integral term, the integral term is attenuated to alleviate the phenomenon of integral windup. Simultaneously, more significant proportional, integral, and differential coefficients are adopted. A slight error and high-gain control method enhance the response effect of minor temperature differences. Considering the respective roles and effects of proportion, differentiation, and integration at different stages of the adjustment process, along with their impact on control system performance, adjusting the controller structure based on the varying deviation values during different stages of the control process proves effective in overcoming the effects arising from solder phase transitions. As a result, the overshoot of the system and settling time are reduced, system convergence is accelerated, and control system performance is improved. Considering the varying heat capacities of different workpieces, introducing an adjustable power upper limit allows for using corresponding laser power upper limits for different workpieces. By setting the maximum power of the output, the amplitude of the controller’s output can be constrained. On the one hand, it ensures a controlled heating rate at the welding point for maintaining welding quality. On the other hand, it reduces the magnitude of the initial state error, thereby resolving the issue of derivative kick. Meanwhile, this enhances the adaptability of laser soldering, reduces dependency on hardware equipment performance, and ultimately lowers costs. The final output value of the laser power can be calculated as follows:

(4) $$u(n)=\left\{\begin{array}{l} P_{0}C_{1}\;\;\;\;\;\;\;\;\;\;\;\;\;\;\;\;u(n)⩾P_{0}C_{1}\\ u(n)\;\;\;\;\;\;\;\;\;\;\;\;\;\;\;\;\;u(n)<P_{0}C_{1} \end{array}\right.$$

where $P_{0}$ is the upper limit of the laser power (0~100%), and $C_{1}$ is the power coefficient, which converts the upper limit of the power into the upper limit of the signal to control the semiconductor laser.

#### 3.1.2. Stability Analysis

**Figure 4:**
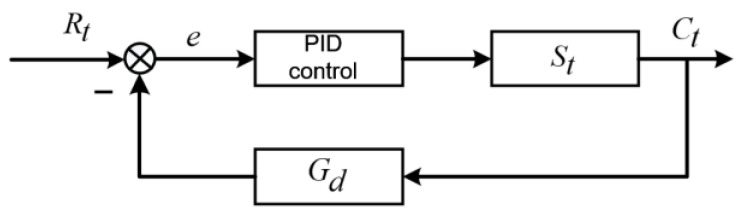
Simplified temperature control loop.

The temperature control loop was simplified to complete the analysis of stability. The temperature control loop was simplified as illustrated in Figure 4.

$R_{t}$ is the target temperature of the temperature control system, and $C_{t}$ is the temperature of the solder joint, which is measured by an infrared thermometer. $G_{vs}$ represents the transfer function of the laser control model, $S_{t}$ represents the control signal coefficients for driving the semiconductor lasers, and $G_{vs}$ is the transfer function of the high-speed solder joint temperature feedback loop. Therefore, $G_{vs}$ can be represented by

(5) $$G_{vs}=\frac{1}{T_{c}s^{2}+2s+1}$$

where $T_{c}$ is the cycle period of the control system, which is short enough to be ignored. Therefore, $G_{vs}$ can be regarded as a consecutive system. $G_{d}$ can be described as

(6) $$G_{d}=\frac{1}{T_{t}s+1}$$

where $T_{t}$ is the cycle period of the infrared thermometer response, which is 1 ms. According to Figure 4, the transfer function of the entire temperature control loop can be described as

(7) $$\begin{array}{cc} G_{s} & =\frac{G_{vs}S_{t}}{1+G_{vs}G_{d}S_{t}}\\ & =\frac{S_{t}(Tts+1)}{(T_{c}s^{2}+2s+1)(T_{t}s+1)+S_{t}}\\ & =\frac{S_{t}(T_{ts}+1)}{T_{t}T_{c}S^{3}+(2T_{t}+T_{c})S^{2}+(2+T_{t})S+1+S_{t}} \end{array}$$

According to the characteristic equation of the control system and the Routh criterion, the stability condition of the control system can be presented as

(8) $$\frac{\left(2T_{t}+T_{c})(2+T_{t})-T_{t}T_{c}(1+S_{t}\right)}{2T_{t}+T_{c}}>0$$

The stability condition of the system can be evaluated as

(9) $$\begin{array}{c} \frac{\left(2T_{t}+T_{c})(2+T_{t}\right)}{T_{t}+T_{c}}>(1+S_{t})\\ S_{t}>0.02 \end{array}$$

The system error can be expressed as

(10) $$E(s)=R_{t}(s)-C_{t}(s)G_{d}(s)=R_{t}(s)\left(1-G(s)G_{d}(s)\right)$$

The system steady-state error can be given in Equation (11),

(11) $$e_{ss}=\underset{s\to 0}{\lim}\;sE(s)=\underset{s\to 0}{\lim}\;sR_{t}(s)\left(1-G(s)G_{d}(s)\right)$$

When the system input is the pulse signal, that is, $R_{t}(s)=R_{t}$, the steady-state error can be described as

(12) $$e_{ss}=\underset{s\to 0}{\lim}\;sE(s)=\underset{s\to 0}{\lim}\;sR_{t}\left(1-G(s)G_{d}(s)\right)=0$$

When the system input is the step signal, that is, $R_{t}(s)=R_{t}(s)/s$, the steady-state error can be described as

(13) $$\begin{array}{cc} e_{ss} & =\underset{s\to 0}{\lim}\;sE(s)=\underset{s\to 0}{\lim}\;s\frac{R_{t}}{s}\left(1-G(s)G_{d}(s)\right)\\ & =\underset{s\to 0}{\lim}\;R_{t}\left(1-G(s)G_{d}(s)\right)=0 \end{array}$$

In summary, according to the Routh criterion, the control system is stable.

### 3.2. Optimization Model of Process Parameters Based on ResNet

During the soldering process, the target temperature for each stage is determined, typically requiring adjustments to the upper limit of the laser power and heating time. The quality is then evaluated based on the surface state of the solder joints and solder splatter. Appropriate upper limits of the laser power and heating time can influence the quality. Traditionally, engineers determine these values through experience and experimentation, resulting in significant time and raw material costs. This paper introduces a ResNet to predict the upper limit of the laser power and heating time, offering a more efficient approach.

#### 3.2.1. Neural Network

**Figure 5:**
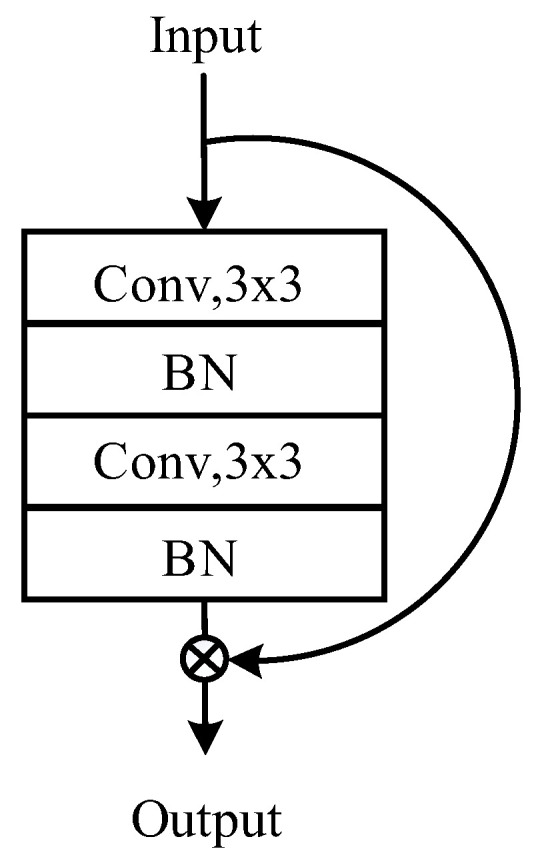
ResNet structure.

The essence of the ResNet lies in its identity shortcut connection, which directly maps low-level features to high-level features, as depicted in Figure 5, which illustrates the ResNet’s network structure. The ResNet addresses the issue of gradient vanishing to some extent by skipping imperfect training layers through the use of identity maps, thereby enabling a deeper network structure. The ResNet is used to analyze the solder joint images to obtain the optimal process parameters. The gradient-weighted class activation mapping (Grad-CAM) technique is employed to visualize the basis of the optimization of the model, thereby gaining a deep understanding of the identification process of the model for the process parameters. Furthermore, a confusion matrix is used to assess the predictive performance of the model, ensuring accuracy and robustness. Through these methods, it is expected to find efficient parameters applicable to practical engineering scenarios.

Grad-CAM builds on CAM by replacing the fully connected layer with global average pooling (GAP) and setting the number of output channels in the final convolutional layer to match the number of categories to be classified [33]. In machine learning, the confusion matrix serves as a classic visual measurement tool. It displays the predictive performance of the model for each category, including True Positive (TP), False Positive (FP), True Negative (TN), and False Negative (FN). Various performance indicators, such as accuracy, precision, and recall values, can be calculated from the confusion matrix to further assess the effectiveness of the model in predicting process parameters.

#### 3.2.2. Transfer Learning

**Figure 6:**
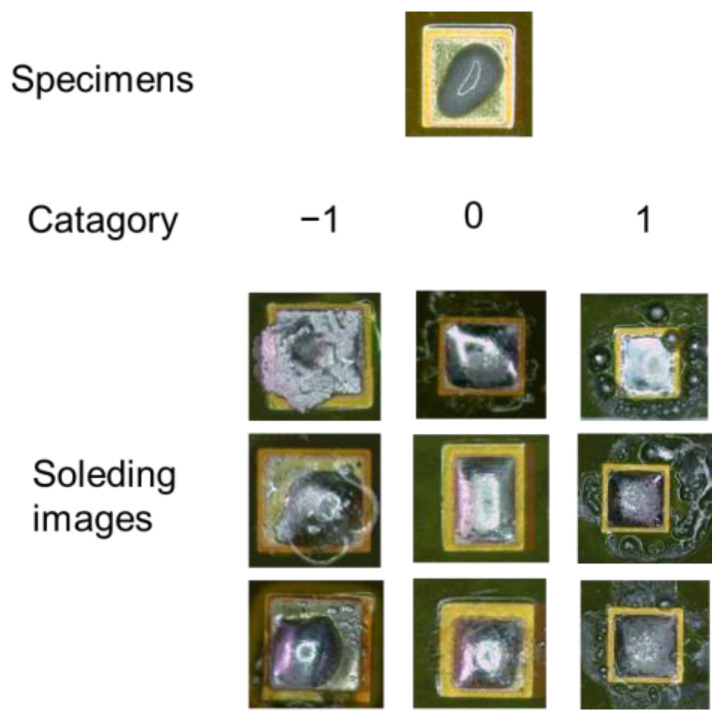
Solder joint classification categories.

As demonstrated in Figure 6, the experimental results show that when the soldering temperatures for each segment are determined, the soldering effect can be classified into three categories: −1, 0, and 1. Category −1 signifies insufficient solder, with some unmelted solder on the pad, indicating a low power upper limit. Category 0 represents the optimal effect, with fully melted solder, round and shiny solder joints, and no apparent solder spattering around the joints, indicating an appropriate laser power upper limit. Category 1 denotes significant solder spatter. Different process parameters can yield similar soldering effects. For the purpose of training the model to discern the relationship between the process parameters and their corresponding results, the initial steps involve conducting soldering tests with a diverse set of process parameters. This is followed by the collection of images representative of varying soldering conditions to serve as training data for the neural network. Images of solder joints are captured using a camera within the optical system.

Experiments have revealed that insufficient heat accumulation results in incomplete solder melting when the soldering time is short or the upper power limit is too low. Conversely, a longer soldering time can cause pad overheating and separation from the substrate, substrate deformation due to heat, and an enlarged heat-affected zone. Excessive heating can damage the workpiece. If the upper power limit is too high, the solder joint temperature will rise too quickly, not allowing enough time for the flux to evaporate. This can lead to flux boiling and vaporization, resulting in solder spatter.

Based on the solder joint morphology, 400 images of solder joints were collected for each heating time. In this dataset, 320 images were utilized as the training set for the purpose of training the algorithmic model, and an additional 80 images were employed as the test set to evaluate the model’s performance and accuracy. Due to the progressive melting process of solder, a significant correlation existed among images from adjacent categories. This correlation hampered the classification process of the neural networks. To reduce this correlation, 20 images were removed from adjacent datasets. Supplementary lighting significantly impacted solder joint shape recognition, as differences in exposure affected the image capture of round and shiny solder joints. To address the overfitting issue arising from a limited number of training samples, 20% of the solder joint information in the sample set was selected and rotated at different angles, with adjusted brightness and contrast, to form new training samples simulating various scenarios. This diversification of the training network input further enhanced the generalization capabilities of the neural networks.

## 4. Experimental Results and Analysis

### 4.1. Experimental Scheme

**Figure 7:**
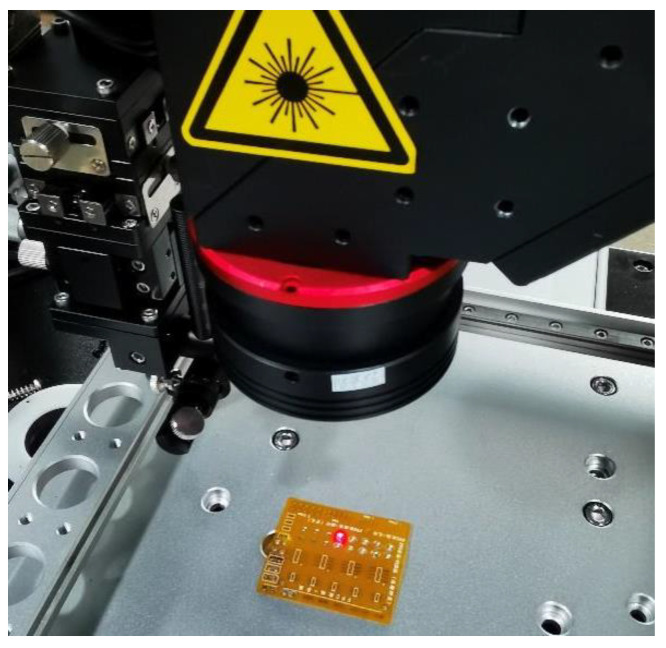
Soldering process.

As shown in Figure 7, soldering experiments were conducted on optical components based on multi-beam coaxial light to verify the reliability of the algorithm. An FPC (with a total thickness of 0.1 mm, a copper foil thickness of 35 μm, and a bottom material of polyimide) was used as the substrate, and the melting point of the solder was 217 °C. The composition is shown in Table 3. The sizes of the two types of solder pads were 0.578 mm^2^ and 1.142 mm^2^, respectively. The temperature curve during soldering was divided into three stages: preheating, soaking, and reflow. The specific process parameters are shown in Table 4.

**Figure 8:**
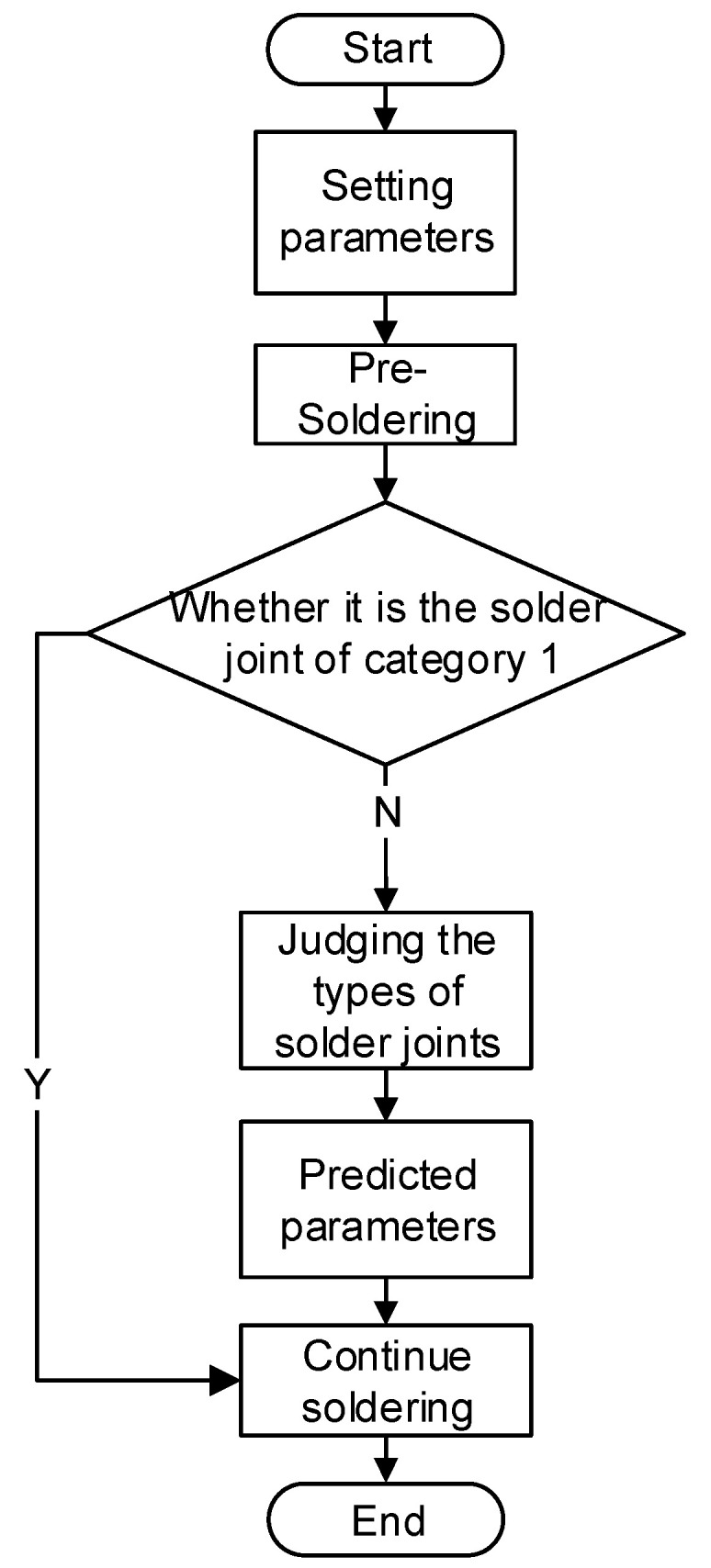
Heating experiment process.

During the experiment, a camera was employed to collect solder joint image information, which was then transmitted to a computer. The trained model was used to identify and classify the images, providing the required process parameters. The entire soldering experiment process is depicted in Figure 8.

### 4.2. Sodering Experiments

**Figure 9:**
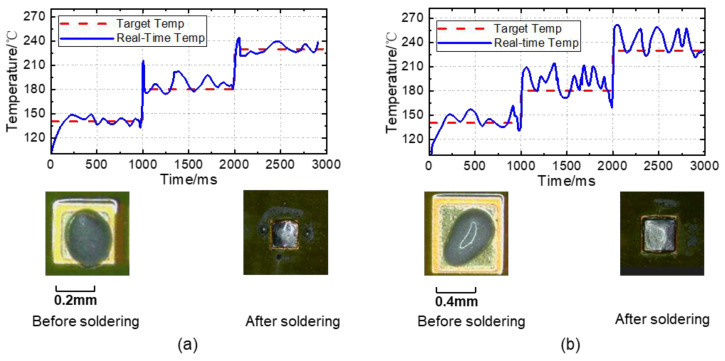
Traditional PID soldering effect and temperature curve. (**a**) Soldering joint with an area of 0.578 mm^2^. (**b**) Soldering joint with an area of 1.142 mm^2^.

Figure 9 displays the soldering experiments for two pads with different areas. In the traditional PID control mode, the temperature adjustment exhibited an overshoot and oscillation defects, whereas the solder joint surfaces underwent significant oxidation. Simultaneously, significant splashing occurred. The main reason for this is that the energy required to heat each workpiece to the same temperature varies and traditional control methods cannot adapt to this change. During soldering, the solder rapidly absorbs heat when transitioning from solid to liquid, and the traditional PID controller cannot effectively adapt to this situation.

**Figure 10:**
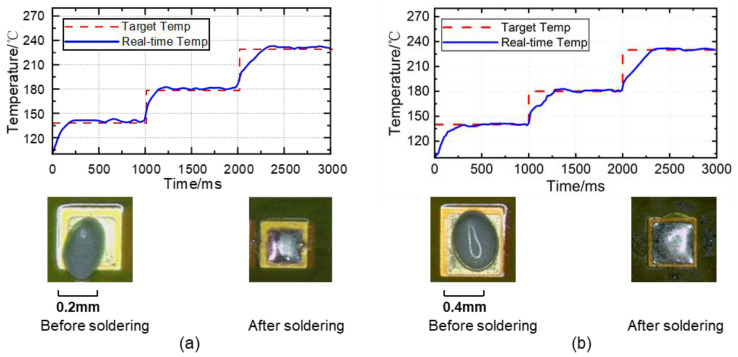
Improved PID soldering effect and temperature curve. (**a**) Soldering joint with an area of 0.578 mm^2^. (**b**) Soldering joint with an area of 1.142 mm^2^. $K_{p}=0.1;K_{i}=0.02;K_{d}=3.5;E_{A}=33$; $E_{B}=9$.

Figure 10 displays the improved variable-structure PID soldering effect and temperature curves. The experimental results indicate a good fit between the temperature of the workpiece and the target temperature, with no temperature overshoot. There was also no phenomenon of integral windup or derivative kick observed. The solder joint had a glossy finish and good wettability, and no noticeable solder spatter around the solder joint was observed. In considering both the temperature fitting effect and the soldering effect, it can be deduced that the control effect of this method is superior to the traditional control method. When dealing with different workpieces, rapid heating can be achieved by setting the laser power upper limit and appropriately increasing the power upper limit when soldering workpieces that are not overly sensitive to temperature, thereby reducing the heating time. The improved control method can dynamically alter the structure of the controller and parameters based on the instantaneous system error, thereby improving the response speed, reducing the overshoot, and enhancing temperature stability performance. This method is particularly effective when soldering precision devices.

**Figure 11:**
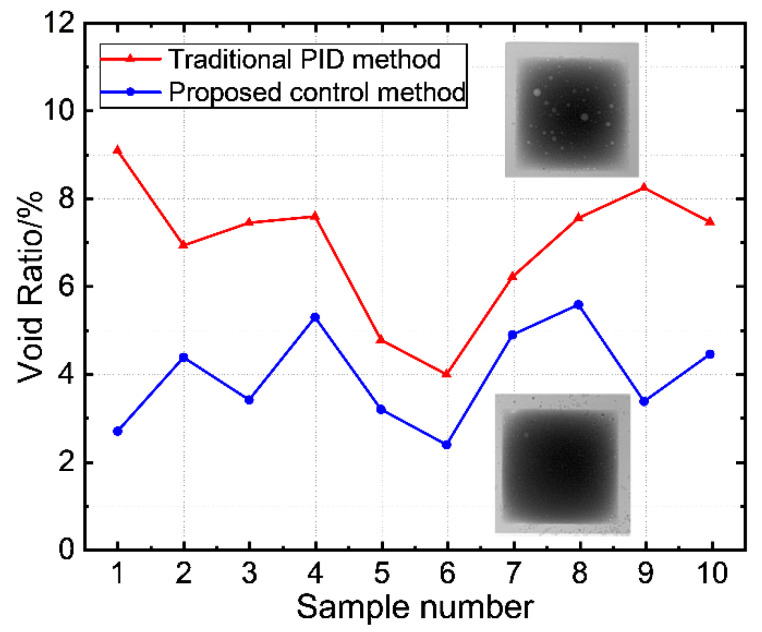
Comparison of solder joint void rates under two control methods.

The flux in the solder is enclosed by liquid metal solder during evaporation so it is unable to evaporate and leaves voids in the solder joint. These voids can negatively impact the physical properties of solder joints, such as thermal and electrical conductivity, leading to decreased mechanical strength in printed circuit boards (PCBs) and increased thermal resistance. Consequently, this reduces the reliability of the soldering [34]. In traditional soldering methods, the rate of the temperature increase in the solder joint is difficult to control, which prevents the effective evaporation of the flux and causes drastic temperature changes. This may result in an increased void rate within the solder joint. As illustrated in Figure 11, X-ray tests on solder joints revealed that under traditional PID control, not only is the surface of the solder joints oxidized but the internal void rate is also significantly increased, with an average void rate exceeding 5%. In contrast, under the proposed improved control algorithm, the internal void rate of solder joints was reduced to less than 6%.

**Figure 12:**
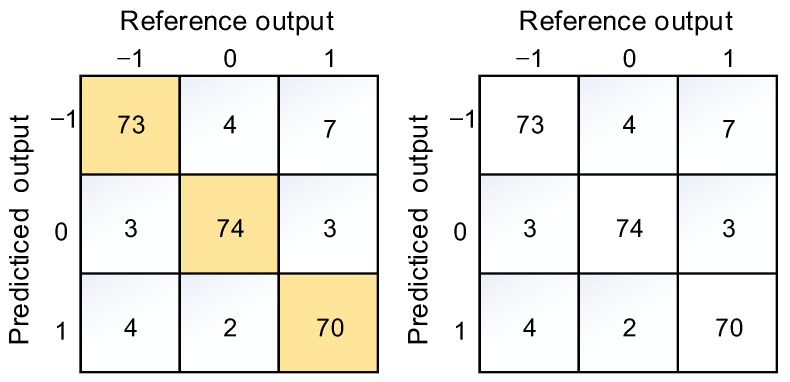
Confusion matrix.

Since the classification result of the soldering image is an ordered categorical variable, experiments have found that adjacent categories can be misclassified. For example, images in category −1 should not be classified as category 0. As shown in the confusion matrix obtained from the experiment in Figure 12, all confusion matrices indicate that the model performed well, with a total accuracy rate of 90.41%. Since the training set and test set images were solder joints from similar categories, there was a specific correlation between their images. After an extended soldering duration, the discrimination of similar solder joints will decrease, but the heat-affected zone range will expand at this time. Suppose the soldering time is too short or the power upper limit parameter is too low. In that case, part of the solder cannot be melted, resulting in spherical solder, which resembles the shape of partially spattered solder.

**Figure 13:**
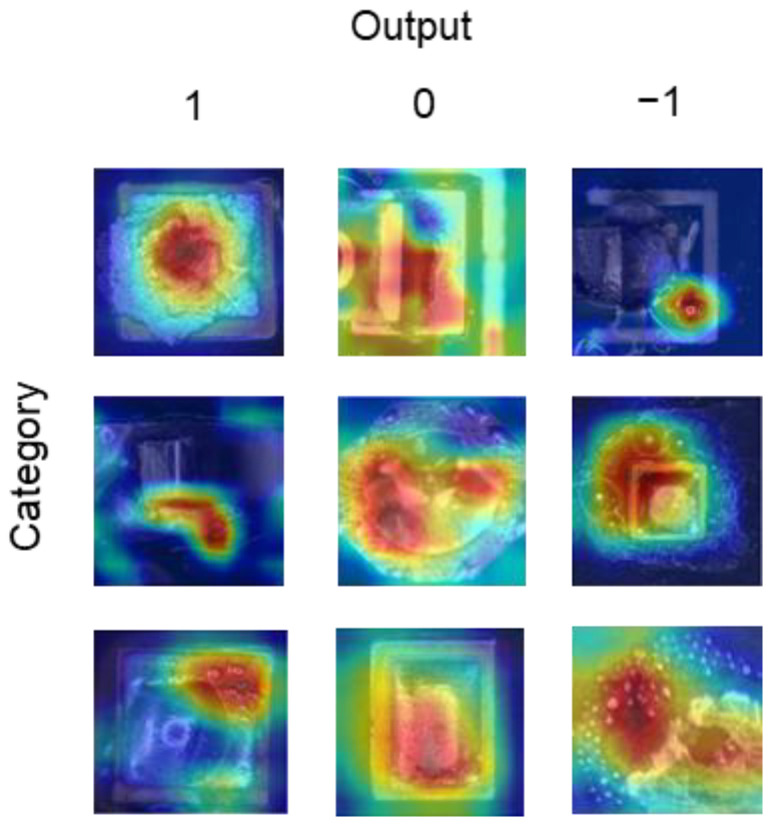
Grad-CAM results of experiment.

The Grad-CAM method was employed to create heat maps to ascertain the image features that the neural network model primarily focused on, providing insights into the intrinsic relationship between the output classes and the input. Figure 13 illustrates the results of the solder joint classification using the Grad-CAM method. As depicted in the figure, the model prioritized the edges of the solder joints and areas with distinct characteristics, such as splattered tin balls or smooth regions on the solder joint surface. These features, which varied according to alterations in the soldering process parameters, served as the basis for distinguishing between the different categories of images. During prolonged soldering, the solder joint and substrate endure prolonged exposure to high temperatures in the air, exacerbating surface oxidation. A higher-power upper limit can cause the solder temperature to surge rapidly in a short time period, and a shorter soldering duration can prevent the internal flux from evaporating in time, leading to splattering during soldering. An insufficient power upper limit or inadequate heating duration can result in insufficient melting of the solder.

**Figure 14:**
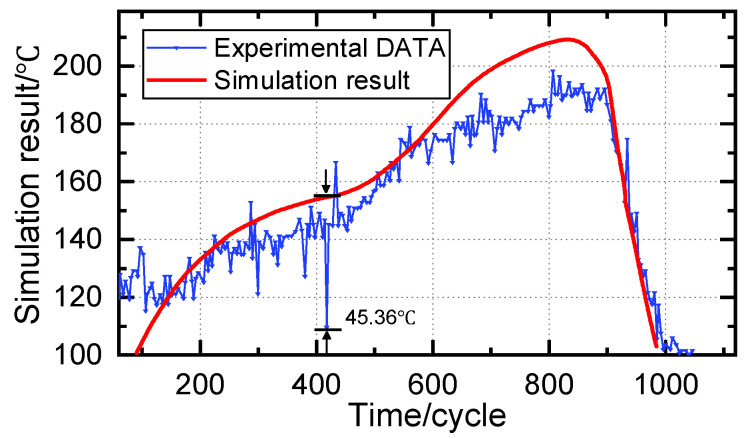
Temperature control performance comparison with Chen’s study.

Figure 14 depicts Chen et al.’s experimental and simulation results [7]. During the heating process, the average temperature error of the solder joint was 17.54 °C, and the maximum temperature error reached 45.36 °C. Compared to this method, the approach under discussion exhibited broader adaptability, a superior correlation between the temperature of the workpiece and the target temperature, and smaller steady-state errors when the temperature was stable.

### 4.3. Exploration of Soldering Quality Factors

Numerous experimental results indicate that the final soldering outcome is closely related to the upper limit of the laser power and heating time. If the heating time is too long or too short, the workpiece and base material can be burned or the solder might not melt completely. An excessively high or low power limit can also cause issues: when the laser energy is too high, the workpiece heats up rapidly, potentially leading to solder splashing. Conversely, when the energy is too low, the target temperature is not reached and the solder remains unmelted.

**Figure 15:**
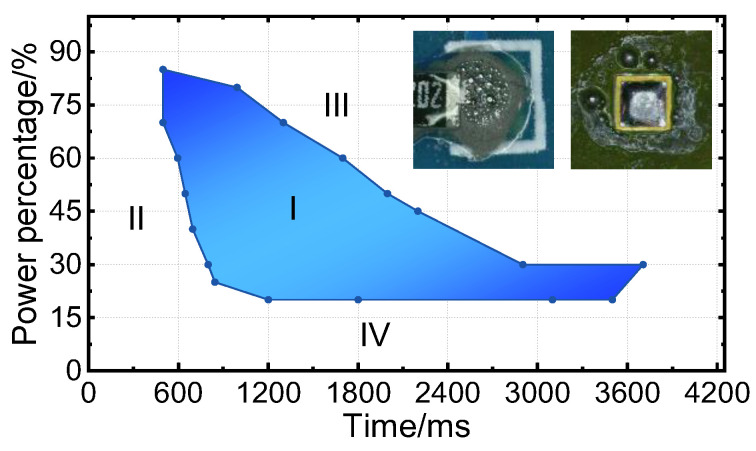
The relationship between the process parameters and soldering quality.

To study this, an orthogonal test was designed to refine the process parameters. A successful solder joint was characterized by a smooth surface with no obvious solder splashes around the pad. Figure 15 displays the final interval obtained.

A favorable outcome, characterized by the absence of spattering and unmelted solder, was observed in Area I. Area II exhibited insufficient heat accumulation due to a short heating time, causing incomplete solder melting. Excessive heat accumulation in Area III was attributed to an overly high power upper limit or extended heating time, leading to solder joint oxidation and solder sputtering. In Area IV, a low power upper limit prevented the melting of the solder. These test results provide a solid foundation for subsequent in-depth research and process optimization.

**Figure 16:**
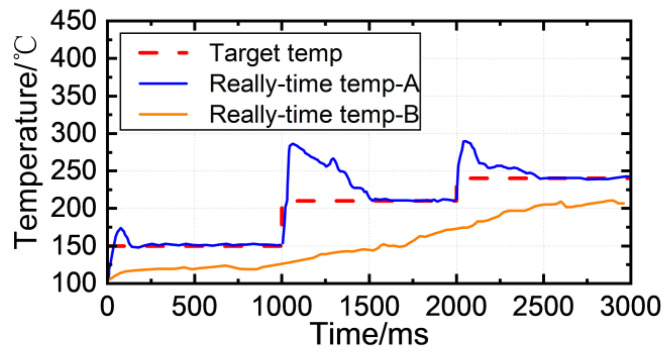
The effect of the upper limit of laser power on the temperature of solder joints.

The upper power limit is critical for the final soldering outcome. Suppose the neural network model produces an incorrect prediction. In this case, it can result in two temperature curves, as shown in Figure 16. The curve “Real-time temp-A” represents the temperature curve when the predicted power upper limit is too high. Even when using the variable-structure PID algorithm without restricting the laser power upper limit, the temperature will overshoot due to a rapid temperature rise rate and an unresponsive controller and laser drive circuit. The curve “Real-time temp-B” corresponds to the temperature curve when the predicted power upper limit is too low, causing the soldering temperature to fall short of the target temperature. Consequently, the solder is inadequately melted, and the soldering outcome is unsatisfactory. In this situation, the soldering effect will resemble a category −1 case.

## 5. Conclusions

Laser soldering technology serves as an effective soldering method for specialized electronic components, with the surface temperature control of solder joints being crucial to the soldering outcome. This paper introduces a variable-structure PID intelligent temperature control algorithm for solder joints based on an adjustable power upper limit. This approach differs from traditional laser soldering methods in that it enhances the control accuracy of the solder joint surface temperature and reduces the time required for setting the process parameters. The variable-structure control exhibits high robustness, and the ResNet efficiently predicts the optimal process parameters by assessing the surface state of the solder joints. The visual classification results offer valuable insights for further model optimization. Additionally, these experimental results provide strong support for real-time monitoring and process parameter optimization of the laser soldering process. The experimental results indicate that this method can effectively improve the control precision, dynamic performance, and robustness of the soldering temperature in the laser soldering system.

## Figures and Tables

**Table 1:** Symbol explanation for the heat distribution formula.

Symbol	Value
A	Absorptivity
$P_{}$	Laser output power
w	Laser beam radius
r	Distance from laser beam center

**Table 2:** Symbol explanation for the control algorithm formula.

Symbol	Value
$K_{p}$	Proportional coefficient
$K_{i}$	Integral coefficient
$K_{d}$	Differential coefficient
e(n)	Error function
u(n)	Output function
$G_{d}(e)(0<G_{d}(e)\leq 1)$	Subtractive function of error e(n)
$G_{i}(e)(0<G_{i}(e)\leq 1)$	Increasing function of error e(n)
$E_{A}$,$E_{B}$	Error value

**Table 3:** Solder SnAg3.0Cu0.5 composition.

Material	Proportion
Sn	96%
Ag	3%
Cu	0.5%

**Table 4:** Process parameters.

Parameter	Value
Preheating temperature	140 °C
Soaking temperature	180 °C
Reflow temperature	230 °C
Soldering temperature Per segment	1000 ms
Power upper limit Per segment	30%

## Data Availability

The data presented in this study are available on request from the corresponding author.
